# A decade of progress providing safe abortion services in Ethiopia: results of national assessments in 2008 and 2014

**DOI:** 10.1186/s12884-017-1266-z

**Published:** 2017-03-04

**Authors:** Yohannes Dibaba, Sally Dijkerman, Tamara Fetters, Ann Moore, Hailemichael Gebreselassie, Yirgu Gebrehiwot, Janie Benson

**Affiliations:** 1 Research and Evaluation Unit, Ipas Ethiopia, Addis Ababa, Ethiopia; 2 Guttmacher Institute, New York, USA; 3 Ipas Africa Alliance, Ipas, Nairobi, Kenya; 4 Faculty of Medicine, Addis Ababa University, Addis Ababa, Ethiopia

**Keywords:** Ethiopia, Safe abortion care, Abortion complications, Public sector, Post-abortion contraception

## Abstract

**Background:**

Ethiopia has one of the highest maternal mortality ratios in the world (420 per 100,000 live births in 2013), and unsafe abortion continues to be one of the major causes. To reduce deaths and disabilities from unsafe abortion, Ethiopia liberalized its abortion law in 2005 to allow safe abortion under certain conditions. This study aimed to measure how availability and utilization of safe abortion services has changed in the last decade in Ethiopia.

**Methods:**

This paper draws on results from nationally representative health facility studies conducted in Ethiopia in 2008 and 2014. The data come from three sources at two points in time: 1) interviews with 335 health providers in 2008 and 822 health care providers in 2014, 2) review of facility logbooks, and 3) prospective data on 3092 women in 2008 and 5604 women in 2014 seeking treatment for abortion complications or induced abortion over a one month period. The Safe Abortion Care Model was used as a framework of analysis.

**Results:**

There has been a rapid expansion of health facilities eligible to provide legal abortion services in Ethiopia since 2008. Between 2008 and 2014, the number of facilities reporting basic and comprehensive signal functions for abortion care increased. In 2014, access to basic abortion care services exceeded the recommended level of available facilities providing the service, increasing from 25 to 117%, with more than half of regions meeting the recommended level. Comprehensive abortion services increased from 20% of the recommended level in 2008 to 38% in 2014. Smaller regions and city administrations achieved or exceeded the recommended level of comprehensive service facilities, yet larger regions fall short. Between 2008 and 2014, the use of appropriate technology for conducting first and second trimester abortion and the provision of post abortion family planning has increased at the same time that abortion-related obstetric complications have decreased.

**Conclusion:**

Ten years after the change in abortion law, service availability and quality has increased, but access to lifesaving comprehensive care still falls short of recommended levels.

## Background

Ethiopia, the second most populous country in Sub-Saharan Africa (SSA) with a population of over 90 million, has recently experienced rapid economic and social changes including improvements in maternal and child health. According to the World Health Organization (WHO), the maternal mortality ratio in Ethiopia has declined from 1400 per 100,000 live births in 1990 to 420 per 100,000 live births in 2013; yet, this level of maternal mortality remains one of the highest in Africa and in the world [1]. Similar to most developing countries, the primary causes of maternal death in Ethiopia include unsafe abortion, hemorrhage, sepsis, obstructed pregnancy, and hypertensive disorders [2, 3]. In 2008, WHO estimated that 18% of maternal deaths in East Africa as compared to 13% globally were caused by unsafe abortions [4]. Unsafe abortion was the highest contributor to maternal death in Ethiopia between 1980 and 1999, accounting for approximately a third of maternal mortality, but it has declined substantially since to about 10% in 2014 [2]. Such a decline in the contribution of unsafe abortion to global maternal mortality has been evidenced by a recent WHO systematic analysis which showed that by 2013 the contribution of unsafe abortion to maternal mortality was 10% in SSA as compared to 7.9% worldwide [5].

To reduce deaths and disabilities from unsafe abortion, the Ethiopian Parliament liberalized its abortion law in 2005 to allow safe abortion under certain conditions. Prior to reform, abortion was prohibited except in cases where the pregnant woman was in grave or imminent danger. Since 2005, abortion is permitted in the following cases: rape or incest; when the pregnancy endangers the woman’s life or health; fetal abnormalities; if the woman is physically or mentally disabled; and if the woman is physically or psychologically unprepared to raise a child due to young age [6]. Following legal reform, the Ministry of Health (MoH) launched the Technical and Procedural Guidelines for Abortion Care in 2006 which led to a rapid expansion of health facilities providing safe abortion services. This facility expansion was supplemented by training health professionals, with a strong focus on mid-level providers. Non-govermental organizations (NGO) such as Ipas and EngenderHealth have partnered with the MoH to support the public health system in the in-service training of providers.

Deaths and disabilities due to unsafe abortion are easily preventable. When induced abortion is performed in sanitary conditions by trained health care providers, it is one of the safest medical procedures performed for women of reproductive age. Despite this, in 2008 almost 58,000 women sought care for complications of abortion in Ethiopia, with 41% experiencing moderate or severe morbidity and a case fatality rate of 628 per 100,000 among women seeking care at public hospitals [7]. Out of an estimated 382,000 induced abortions in 2008, only 27% were legal procedures performed in a health facility [8]. By 2014, the proportion of legal abortions that took place in health facilities increased to 53%, despite the number of induced abortions increasing to about 622,000 [9]. These showed that improving accessibility and provision of safe abortion care and post-abortion family planning is important to prevent maternal mortality and morbidity from unsafe abortionin Ethiopia.

In spite of the known contribution of unsafe abortion to maternal mortality, there have not been studies to examine the capacity of the health system to provide comprehensive abortion care (CAC). To address this gap, this study aimed to measure Ethiopia’s progress toward scaling up CAC services following the legal reform by conducting national assessments in 2008 and 2014. Data from two points in time allow us to describe how changes in legal reform and government interventions have impacted the availability and utilization of safe abortion services between 2008 and 2014. This paper builds on the work done by Abdella and colleagues [10], who applied the Safe Abortion Care (SAC) model [11] to measure indicators of safe abortion care in 2008 to provide baseline levels of SAC services in the country soon after legal reform. For this paper, we repeated the analyses conducted by Abdella and colleagues, comparing results from 2008 to 2014.

### Model for measuring availability and utilization of safe abortion services

We draw on Healy and colleagues’ process indicators for emergency obstetric care (EmOC) for measuring three essential elements of safe abortion care [11]. These include provision of induced abortion, treatment of abortion complications, and post-abortion contraception. As with the EmOC model [12] the Safe Abortion Care (SAC) model similarly assumes that the availability of abortion services which are geographically dispersed, of high quality and well utilized will lead to the decline of abortion-related mortality and morbidity [11].

The SAC model includes a set of signal functions and seven abortion indicators for monitoring the availability and use of abortion care services. The signal functions are essential services that health facilities must have available and functioning in order to ensure delivery of quality SAC and are categorized by the type of SAC (basic or comprehensive) services expected to be provided by a facility. Facilities providing basic SAC, generally health centers or smaller primary level health facilities, are expected to offer first trimester abortion care and are expected to perform the following six signal functions: (1) perform induced abortion, or termination of pregnancy (TOP), for uterine sizes up to 12 weeks for all legal indications; (2) provide post-abortion contraception; (3) administer essential antibiotics; (4) administer intravenous replacement fluids; (5) administer oxytocics; and (6) perform removal of retained products of conception for uterine sizes up to 12 weeks, or post-abortion care (PAC). Facilities offering comprehensive SAC, generally higher level facilities or hospitals, are expected to offer first and second trimester abortion care. Comprehensive SAC includes the six signal functions required for basic SAC plus an additional four : (1) perform induced abortion (TOP) for uterine sizes greater than 12 weeks for all legal indications; (2) perform removal of retained products of conception (PAC) for uterine sizes greater than 12 weeks; (3) perform blood transfusion; and (4) perform laparotomy. Induced abortion is expected to be available during regular outpatient hours, while the nine remaining signal functions should be available 24 h per day, 7 days per week [10, 11].

The SAC model’s seven indicators for abortion care include two indicators for monitoring service availability and geographic distribution and five indicators to measure progress in service utilization and quality [11]. For service availability and geographic distribution, the model recommends five health facilities per 500,000 residents, four of which should offer basic and one comprehensive SAC [11, 12]. The model recommends that 100% of sub-national areas have an adequate level of SAC per the recommendation indicated above. The five indicators of service utilization and quality are calculated using facility service statistics and include the proportion of women treated for obstetric complications that are abortion-related, the proportion of abortion complications that are serious, the proportion of abortion services that are induced abortion procedures, the proportion of uterine evacuations performed with appropriate technology, and the proportion of women receiving abortion services who obtained contraception. For more detailed information on the SAC model, see Healy et al. [11].

## Methods

Data used for this study come from nationally representative cross-sectional health facility studies conducted in Ethiopia in 2008 and 2014 as part of a national study on abortion incidence and morbidity. Two of the data sources used for determining abortion incidence and morbidity were used for this analysis: a national cross-sectional Health Facility Survey (HFS) and a Prospective Morbidity Survey (PMS). Data collection for the 2008 study occurred between November 2007 and February 2008, and the full methodology is described and published in Gebreselassie et al. [7] and Singh et al. [8]. The 2014 study data were collected from December 2013 to April 2014 and the methodology is described in detail by Gebrehiwot et al. [9, 13]. In both 2008 and 2014, the health facility survey included log-book reviews to collect data on service utilization and quality required for the SAC model.

Ethical approval was obtained from the National Health Research Ethics Review Committee at the Ministry of Science and Technology of Ethiopia, Addis Ababa and from the Guttmacher Institute ethical review committee. Informed oral consent was obtained from every participating patient in the PMS and from the health providers in the HFS.

### Sampling of health facilities

To construct the sampling frame, a list of health facilities was obtained from the Ministry of Health in 2008 and the Food, Medicine and Health Care Administration and Control Authority of Ethiopia (FMHACA) in 2014. This list included public hospitals, public health centers, private hospitals, private higher clinics, and NGO reproductive health clinics. Private medium clinics were included in 2014, but not in 2008. In both years, health facilities were eligible for inclusion if they were authorized to provide treatment for abortion complications or induced abortion services, according to the Technical and Procedural Guidelines for Abortion Care [6, 14]. The sampling frame consisted of 896 eligible facilities in 2008 and 4287 facilities in 2014.

Multi-stage stratified random sampling was used. The first strata, region (*n* = 11), and the second, facility type and ownership, were used (33 in 2008 and 77 in 2014) to draw a nationally and geographically representative sample using probability-based sampling. Higher level facilities known to provide abortion and maternity-related services such as hospitals were sampled at a higher fraction, while health centers and medium private facilities were sampled at a relatively lower fraction. Sampling fractions ranged between 0.12 and 1.00 of all eligible facilities.

### Data sources

As mentioned above, two data collection methods and tools were used to measure facilities’ capacity to provide safe abortion services both in 2008 and 2014: the Health Facility Survey and the Prospective Morbidity Survey.

#### Health Facility Survey (HFS): interview and logbook review

For the HFS, trained data collectors visited each selected facility and interviewed the most senior abortion care provider or someone knowledgeable about induced abortion and post-abortion care services. Respondents included obstetrician/gynecologists, general practitioners, midwifes, nurses, and other health workers. The HFS interviewers obtained information on the facility infrastructure, the facility’s capacity to provide abortion services, type of abortion care provided and monthly caseload of abortion patients, provider attitudes towards abortion service provision, and the facility’s ability to perform each of the EmOC and SAC signal functions during the previous 3 month period.

The HFS interviewers also recorded logbook information on cases of abortion and obstetric complications recorded at each facility for the previous year. Collected information from the facilities’ abortion logbooks included the number of abortion cases, severity of abortion complications, the abortion method used, and provision of post-abortion contraception. Following the method used in Abdella et al. [10] logbook review captured women seeking an induced abortion, and women treated for complications of miscarriage or abortion induced outside the health facility and presenting for treatment. The interviewers also collected logbook data on the number of obstetric complications including hemorrhage, sepsis, prolonged or obstructed labour, complications from abortion, pre-eclampsia or eclampsia, ectopic pregnancy or ruptured uterus. The HFS data were assigned the appropriate facility weight. A total of 335 facilities in 2008 and 822 facilities in 2014 participated in the HFS, with participation rates of 96 and 89% for public hospitals and health centers respectively in 2008, and 98 and 93% respectively in 2014.

#### Prospective Morbidity Survey (PMS)

For the PMS, data were collected prospectively on all women presenting with abortion complications or requests for a legal induced abortion during a consecutive 30-day period in each study facility. Providers in the facilities were trained to record information on each woman requesting a safe induced abortion or seeking PAC either for complications of an unsafe abortion or miscarriage. Data were also recorded on patient demographics, self-reported induction attempts, reproductive history, vital signs, symptoms found on physical examination, abortion symptoms that drew the woman to the facility, and essential elements of the care provided to her. Patients were not interviewed directly on these questions—it was information provided as part of patient intake and treatment.

A total of 344 facilities in 2008 and 569 facilities in 2014 participated in the PMS. The PMS included data on 3092 women in 2008 and 5604 women in 2014. More details on the sampling, weighting, data collection tool, and participation rate of the facilities for both the HFS and PMS have been published in Moore et al. [7] and Gebrehiwot et al. [13].

### Data analysis

Data from the 2008 and 2014 studies were entered and checked for consistency and completeness using EpiData version 3.1. Datasets from both years were imported into, cleaned, and analyzed both separately and combined using Stata version 13.1. Each of the strata were then weighted for non-response and their probability of selection to achieve national representation. All analyses accounted for the complex sample design and appropriate survey weighting, and are presented by year and by facility type (hospitals or health centers). Missing data were limited, and so analysis was limited to non-missing responses. Analysis techniques conducted and described in Abdella et al. [10] were repeated to reanalyze the 2008 data from public sector facilities only, disaggregating by facility type. The Health Facility Survey sample from both years, unweighted and weighted frequencies as well as weighted proportions, are presented by public facility type in Table 1. Ninety public hospitals and 152 public health centers were included in the sample in 2008; 117 hospitals and 368 health centers were included in the sample in 2014.

**Table 1:** Distribution of 2008 and 2014 sample in Ethiopia by public facility type^a, b^

	2008			2014		
Facility type	Unweighted *n*	Weighted *n*	%	Unweighted *n*	Weighted *n*	%
Public hospitals	90	94	13.8	117	120	4.4
Public health centers	152	587	86.2	368	2596	95.6

^a^Percentages are weighted to be nationally representative

^b^Results differ slightly from those published in Abdella et al., [10] due to reanalysis

Performance of basic and comprehensive SAC signal functions were analyzed descriptively. Weighted frequencies and proportions of public hospitals’ and health centers’ reported performance of each signal function are presented. Basic SAC delivery was calculated including all public facilities, and comprehensive SAC delivery was limited to public hospitals only. Safe abortion indicators and their 95% confidence intervals are presented by facility level using weighted frequencies and percentages from both the HFS and PMS data. The SAC model and estimates of 2008 and 2014 Ethiopia regional population sizes using the latest census and projections [15, 16] were used to calculate recommended levels of basic and comprehensive SAC delivery in both years, by region and nationally. Weighted frequencies of basic and comprehensive SAC delivery were used to calculate the percentage of the recommended level of service delivery achieved for both service types.

In both years, log book review data from the HFS were used to calculate obstetric complications, estimates of either the numerator or denominator, were collected only on the HFS; the rest of the indicators come from the PMS which were used to collect client level data on each woman’s presentation, treatment and care. In 2008 only, data on post-abortion contraception was only collected in the HFS. For these indicators, the HFS and PMS datasets were combined, and the HFS estimates were scaled to match the PMS estimate of the scale of difference between variables in the two datasets. The final SAC indicator estimates are presented as percentages of the weighted frequencies along with confidence intervals.

Appropriate technology indicators were defined according to WHO recommendations – vacuum aspiration and medical abortion in the first trimester and dilatation and evacuation (D&E) and medical abortion in the second trimester – with the exception of D&E in the second trimester [17]. This decision was made because even by the 2014 study, no clinical trainings on D&E had been provided for abortion providers in Ethiopia, and the supplies for this procedure are not available in the country. Yet, D&E is often used to describe procedures using sharp curettage, a technology which is not recommended by the WHO [17].

## Results

Since legal reform in 2005, there has been a rapid expansion of public sector health facilities eligible to provide legal abortion services. The number of public facilities eligible to provide basic SAC more than quadrupled (from 587 in 2008 to 2597 in 2014, Table 1). The number of hospitals eligible to provide comprehensive SAC increased from 94 in 2008 to 120 in 2014 (Table 1). As the public health infrastructure in Ethiopia grew over the past decade, so did the capacity of these facilities to perform lifesaving services for basic and comprehensive abortion care.

### Performance of basic and comprehensive SAC signal functions

**Fig. 1:**
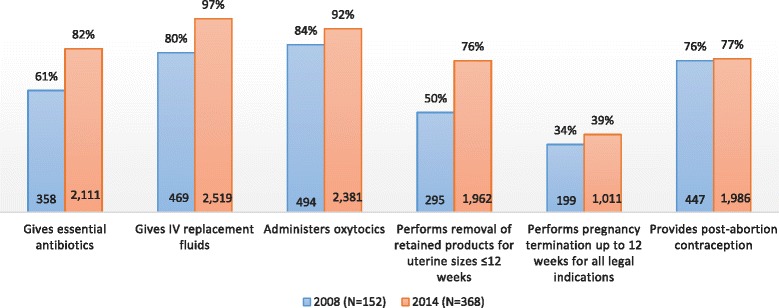
Percent and Frequency of Public Health Centers performing basic SAC signal functions

**Fig. 2:**
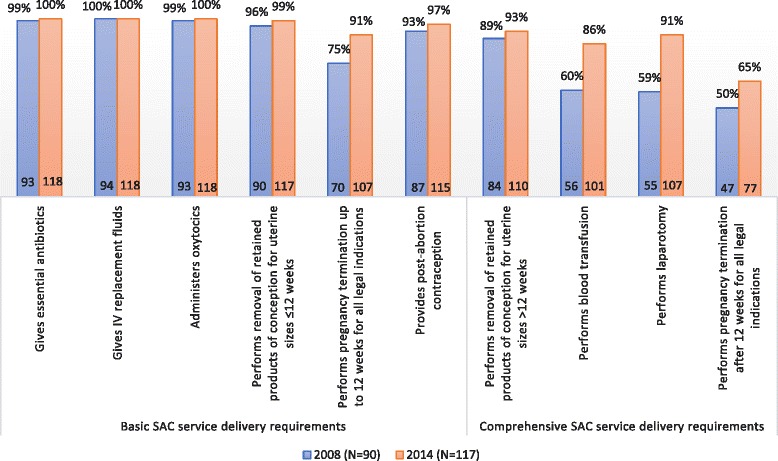
Percent and frequency of Public Hospitals performing basic and comprehensive SAC signal functions

Three years after legal reform (2008), access to basic SAC services varied greatly between public hospitals and health centers, as measured by performance of basic SAC signal functions in the past three months. Performance of basic SAC services was poor in public health centers in 2008. Only one-third of these facilities were able to perform first trimester induced abortion and only half were able to provide PAC services for women in the first trimester of pregnancy (Fig. 1). As expected, basic SAC performance was high in public hospitals in 2008, with 75–100% of hospitals performing each of the six basic signal functions (Fig. 2).

Performance of basic signal functions for SAC improved for both health centers and hospitals between 2008 and 2014. Public health centers showed the greatest improvement in capacity to perform discrete basic signal functions. The proportion of health centers administering parenteral antibiotics increased from 61 to 82%. Almost all public health centers in 2014 reported administering intravenous replacement fluids (97%), an increase from 80% in 2008. The number of public health centers able to administer oxytocics increased nearly five fold, from 469 health centers in 2008 to 2519 in 2014. There was also great improvement in the number of public health centers performing first trimester PAC and first trimester induced abortions, increasing more than six fold and five fold, respectively.

Although there was a large increase in the number of facilities performing first trimester PAC and induced abortion since 2008, still only 76 and 39% of health centers, respectively, provided these essential lifesaving services in 2014. Similarly, while the number of public health centers providing post-abortion family planning (PAFP) increased from 447 to almost 2000 facilities in 2014 making family planning much more broadly accessible, the percentage of health centers providing PAFP only increased from 76% in 2008 to 77% in 2014. Overall, great improvements have been made in both the number and proportion of public health centers performing six signal functions for basic SAC delivery (Fig. 1).

The public sector has also made great progress in preparing hospitals to provide basic SAC, with over 90% of public hospitals now able to perform each of the six signal functions in 2014. The number of public hospitals performing four additional signal functions for comprehensive SAC delivery, their expected level of care, also increased considerably since 2008. Hospitals capable of removing retained products of conception for second trimester pregnancies increased from 89% in 2008 to 93% in 2014. The percentage of public hospitals performing blood transfusion rose from 60 to 86% and the percentage able to perform laparotomy increased from 59 and 91%. Public hospitals able to perform second trimester induced abortion increased from only half of hospitals in 2008 to two-thirds in 2014.

### Achievement of recommended SAC provision

The SAC model recommends five health facilities (four basic and one comprehensive) per 500,000 residents at the national and sub-national levels for service availability and geographic distribution. Based on an estimated population size of 74 million in 2008 and over 87 million in 2014 (15, 16) a minimum of 704 facilities in 2014 must be providing basic SAC services for all women in Ethiopia to have access to this care, compared to a minimum of 591 facilities in 2008 (Table 2). Nationally, the number of public health facilities providing basic SAC increased over five fold, growing from 149 facilities (25% of the recommended level) in 2008 to 823 facilities (117% of the recommended level) in 2014. All but one region, Harari, showed an increase in the number of public facilities providing basic SAC. The number of basic SAC facilities in Harari decreased from three to one. The greatest increase was seen in Oromia, where the number of public facilities providing basic SAC increased from 58 in 2008 to 231 in 2014, yet the region did not achieve the recommended level meeting 87%. Somali, Harari, Afar, and Oromia regions, and the city administration of Addis Ababa fell short of the recommended level in 2014, achieving only 21–92% of the recommended level, yet all but Harari still improved from 2008. The other six regions surpassed the recommended level of basic SAC service delivery in 2014.

**Table 2:** Achieved levels of basic and comprehensive safe abortion care at public facilities nationally and by region in Ethiopia, 2008 & 2014

	Basic SAC service delivery^a^						Comprehensive SAC service delivery^a^					
	2008			2014			2008			2014		
	Recommended^b^	Actual^c^	%	Recommended^b^	Actual^c^	%	Recommended^b^	Actual^c^	%	Recommended^b^	Actual^c^	%
National^d^	591	149	25	704	823	117	148	29	20	176	66	38
Harari	1	3	300	2	1	50	1	0	0	1	2	200
Dire Dawa	3	1	33	3	4	133	1	1	100	1	1	100
Gambella	2	4	200	3	8	267	1	0	0	1	1	100
Addis Ababa	22	1	5	26	21	81	5	6	120	6	7	117
Tigray	35	14	40	40	68	170	9	5	56	10	10	100
Oromia	217	58	27	263	241	92	54	6	11	66	23	35
Amhara	138	38	28	160	231	144	34	5	15	40	9	23
SNNPR	120	26	22	143	218	152	30	6	20	36	8	22
Benshangul-Gumuz	5	1	20	8	11	138	1	0	0	2	0	0
Afar	11	1	9	13	12	92	3	0	0	3	1	33
Somali	36	3	8	42	9	21	9	0	0	11	3	27

^a^Basic or comprehensive status is based on signal function performance in a facility during the previous three-month period as reported by key informants

^b^Recommended level of basic and comprehensive SAC service delivery estimated per population using SAC model (Healy et al., [11])

^c^Frequencies are weighted to be nationally representative

^d^National frequency may not equal the sum of regional estimates due to rounding

Similarily, a minimum of 148 hospitals in 2008 and 176 hospitals in 2014 must provide comprehensive SAC services in order to meet the recommended levels. The number of public hospitals providing comprehensive SAC nationally increased from 29 hospitals in 2008 to 66 in 2014. Despite this increase, the number of public hospitals providing comprehensive SAC still falls far short of the recommended level of 148 in 2008 and 176 hospitals in 2014 -attaining only 20% in 2008 and 38% in 2014 of the recommended levels. The number of public facilities providing comprehensive SAC also increased in every region except two, Benshangul-Gumuz and Dire Dawa, which maintained the same number of facilities providing comprehensive care between 2008 and 2014.

Regional disparities in access to comprehensive SAC have persisted between 2008 and 2014. Whereas smaller regions and city administrations (Harari, Dire Dawa, Gambella, Addis Ababa and Tigray) achieved or exceeded the recommended level of comprehensive service facilities in 2014, larger regions (Oromia, Amhara, SNNPR, Benshangul-Gumuz, Afar, and Somali) fell far short, meeting only 0–35% of the recommended level.

### Indicators of progress in safe abortion care

Table 3 presents indicators for safe abortion care by public facility type in 2008 and 2014. The percentage of all women treated for obstetric complications that were getting treatment for abortion–related care decreased in both public hospitals and health centers, with an overall change from 47% in 2008 to 39% in 2014. This trend was most visible at public hospitals, with women obtaining abortion-related care decreasing from 43 to 31% of women treated for obstetric complications.

**Table 3:** Indicators for safe abortion care by facility type and nationally in Ethiopia, 2008 & 2014^a^

	2008^b^			2014		
	Public Hospitals (*n* = 90)	Public Health Centers (*n* = 152)	Total (*n* = 242)	Public Hospitals (*n* = 117)	Public Health Centers (*n* = 368)	Total (*n* = 485)
	% (95% CI)	% (95% CI)	% (95% CI)	% (95% CI)	% (95% CI)	% (95% CI)
Percentage of women treated for obstetric complications that are abortion related^c^	43 (43, 44)	51 (50, 52)	47 (46, 48)	31 (29, 33)	44 (35, 49)	39 (33, 42)
Percentage of women treated for abortion complications that are serious^d^	30 (28, 31)	29 (26, 31)	29 (27, 31)	22 (20, 24)	37 (33, 40)	32 (29, 34)
Percentage of women who received abortion services that were induced procedures^d^	23 (21, 24)	40 (32, 45)	32 (27, 36)	44 (38, 48)	55 (50, 59)	52 (47, 55)
Percentage of uterine evacuations performed with appropriate technology, regardless of trimester^d,e^	45 (42, 46)	54 (47, 59)	50 (45, 54)	93 (91, 94)	94 (92, 95)	93 (92, 95)
Percentage of uterine evacuations performed with appropriate technology, first trimester^d,e^	61 (58, 63)	62 (55, 68)	62 (56, 66)	96 (95, 96)	95 (93, 96)	95 (93, 96)
Percentage of uterine evacuations performed with appropriate technology, second trimester^d,e^	20 (17, 22)	18 (12, 21)	19 (15, 22)	86 (83, 87)	84 (79, 86)	85 (82, 87)
Percentage of women who received abortion services that obtained contraception^f^	39 (34, 42)	65 (62, 67)	54 (50, 56)	76 (74, 77)	78 (75, 80)	76 (75, 79)

^a^Percentages were calculated using frequencies weighted to be nationally representative

^b^2008 results differ slightly from those published in Abdella et al., [10] due to reanalysis

^c^In both years, denominator from HFS, scaled to match PDS; numerator from PDS

^d^In both years, numerator and denominator from PDS

^e^WHO recommendations for appropriate technology are vacuum aspiration and medical abortion in the first trimester and dilatation and evacuation and medical abortion in the second trimester. Cases missing gestational age were excluded from analysis (*n* = 52 (1%) in 2008 and *n* = 255 (4%) in 2014)

^f^In 2008, numerator from HFS scaled to match PDS; denominator from PDS. In 2014, both numerator and denominator from PDS

A woman is said to have a serious abortion complication if one or more of the following signs are present: death, shock, pulse higher than 119 beats per minute, generalized peritonitis, evidence of mechanical injury or foreign body, organ or system failure, or a temperature higher than 37.9 °C. Nationally, the percentage of women treated for abortion complications that were serious increased slightly from 29 to 32% in the time between the two study periods. The majority of the increase of cases treated for severe complications was in health centers, with an increase from 29 to 37%. Public hospitals actually saw a decrease in this indicator, declining from 30% of women treated for abortion complications in 2008 to 22% in 2014. However, the overlapping confidence intervals for these estimates suggest that these changes were not significant.

The proportion of women who received abortion services that were induced procedures as opposed to PAC increased among both facility types, from 32% in 2008 to 52% in 2014, indicating that more women in 2014 were informed about how to access a safe and legal abortion than the previous time period. The percentage of uterine evacuations performed with appropriate technology regardless of trimester increased from 50 to 93%. Almost all (95%) uterine evacuations in the first trimester were performed with the appropriate technology, vacuum aspiration or medical abortion, at both hospitals (96%) and health centers (95).

Both facility types showed a sharp increase in use of appropriate technology for second trimester uterine evacuations (UE), increasing from 19% in 2008 to 85% in 2014, leaving slightly less than one sixth of second trimester UE procedures being conducted with technology not recommended by the WHO. This trend occurred at the public hospital and health center level, in spite of the fact that health centers are not expected to provide this type of care and are meant to refer second trimester clients to tertiary facilities. Provision of PAFP increased in both facility types between 2008 and 2014: whereas in 2008 slightly over half of women getting abortion services received PAFP, in 20014, approximately three-quarters of women obtained contraception after receiving abortion services (Table 3).

## Discussion

Since legal reform in 2005, health system and community interventions spearheaded by the Ethiopia Ministry of Health have made significant progress in increasing the reach, availability, utilization, and quality of safe abortion care throughout the country. The MoH has worked with a range of donors and development partners, including NGOs such as Ipas and EngenderHealth, in order to make high quality CAC services available at all levels of the public health system. Their efforts have included the development and dissemination of evidenced-based national standards and guidelines in 2006 [6] and a revision in 2014 to incorporate recent WHO recommendations [14]; training of health professionals at all levels of the health system with a particular focus on task-shifting to midlevel providers; integrating SAC and post-abortion contraception into existing reproductive health services; ensuring the sustainable supply of equipment and medications required for SAC; engaging with the private sector to expand their capacity to provide SAC services; and strengthening monitoring and evaluation systems to ensure adherence to quality standards. We focused on public sector facilities because public health facilities serve the overwhelming majority of the population in Ethiopia.

In the six years since the baseline study, there was a rapid expansion of health centers in Ethiopia, which has greatly increased the number of facilities eligible to provide lifesaving SAC services to women. Nationally this has translated into over five fold increase in the number of facilities providing basic SAC, the first level of necessary care, with six regions exceeding the recommended level of basic SAC service delivery. With this increase there has also been a significant shift in where women are accessing safe abortion services. Whereas in 2008 the majority of women (60%) sought SAC services at NGO or private health facilities, by 2014 the majority (56%) were seeking these services in the public sector [13]. The increases in signal function performance are significant achievements, as these services are providing essential life saving care for women with complications for unsafe abortion, women seeking to safely terminate a pregnancy, and women seeking to prevent a future unplanned pregnancy.

Despite these significant increases in availability of basic SAC services, a considerable proportion of health centers are still failing to provide induced abortion services for women presenting in the first trimester, and a quarter of health centers are not providing lifesaving post-abortion care for incomplete abortion and contraceptive services. Regional disparities also persist with five regions not meeting the recommended level of facilities. Regions with the highest proportions of health centers not performing first trimester induced abortion included Afar, Gambella, and Oromia, with over 70% of health centers in each region not performing this service in the preceding three months. Providers at nonperforming facilities most often cited a lack of trained staff, equipment, and supplies as the reasons for nonperformance. It is clear that although great strides have been made in increasing the availability of SAC at health centers, the rapid expansion of these facilities has outpaced the public sector’s preparation of these facilities to provide basic SAC. Improving provision of first trimester PAC and induced abortion and post-abortion contraception should be highlighted as the greatest priority for future health systems interventions at the primary facility level, with particular focus on the regions falling behind.

The vast development of primary health centers in Ethiopia has not been mirrored at the tertiary facility level, although 26 public hospitals have been newly built since 2008. This is unsurprising considering the greater costs and staffing challenges associated with building a new hospital. However, as the population continues to grow at such a pace, so too will the demand for comprehensive SAC services and thus the need for more hospitals. As a result, although hospitals made great improvements in the performance of all ten signal functions for comprehensive care, there is still a large unmet need for comprehensive SAC services nationally and in about half of the regions. Currently, comprehensive abortion care is concentrated in city administrations and small regions of Ethiopia, leaving the majority of women in larger regions, such as Oromia and Amhara, with limited access to this life-saving care. This problem is particularly distressing in Afar and SNNPR, where over half of public hospitals are not performing second trimester induced abortion services. As health centers are not currently permitted in the Standards and Guidelines to provide second trimester SAC, building more hospitals and preparing these facilities to provide this service is necessary to address this gap in care. Continued efforts to provide comprehensive contraceptive services in order to prevent unplanned pregnancies are equally important.

The regional disparities in provision of safe abortion care that existed in 2008 have persisted. Even in regions that met the recommended level of facilities in 2014, this achievement does not necessarily translate into women being able to access these services, particularly for widely dispersed and remote regions such as Gambella, Afar, and Somali. Because the recommended level in the SAC model is based on population size and does not account for population density or geographical differences between areas, even if the region met the recommended level, women in rural areas or large regions with small populations will need to travel long distances to access abortion care. Meeting the recommended level may decrease the distance they must travel, but this is not universally true as the model does not account for the clustering of facilities in urban areas.

Perhaps the greatest indicator of the public sector’s progress is the declining proportion of women seeking treatment for complications of unsafe abortion (PAC). The proportion of women seeking a safe and legal induced abortion increased from one-third in 2008 to over half of women in 2014, indicating that women are accessing care earlier instead of attempting to induce abortion outside of a health facility. The increase in the provision of post-abortion contraception, an essential step for preventing repeat unintended pregnancy, is also impressive, with over three-quarters of women accepting a method in 2014. This achievement is particularly dramatic considering that only four in ten married Ethiopian women were using modern contraceptive methods in 2014 [18]. In addition, many more women in 2014 were provided abortion care using WHO-recommended technologies, with improvements in both first and second trimester care. This is mainly due to the near elimination of the use of sharp curettage for first trimester procedures, a more expensive and painful method with higher complication rates than MA and MVA. The same positive trend is required for second trimester abortion using WHO recommeneded technologies. As the revised technical and procedural guideline by the MOH emphasizes second trimester abortion as an important component of CAC services, allowing additional cadre of providers such as integrated emergency surgical officers (IESO) to provide second trimester services [14], increasing the training of providers in second trimester abortion service is essential to fill this gap.

Abortion complications as a proportion of obstetric complications have decreased since 2008. The same trend was documented among hospitals in an evaluation of a SAC intervention in the Tigray region from 2006 to 2009 [19]. Because abortion-related morbidity and mortality are so difficult to study and measure accurately, it is important to recognize this impressive reduction of obstetric complications related to abortion. On the other hand, there was no significant change in severe complications, which is consistent with the findings by Gebrehiwot et al. who found little change in severe complications since 2008 [13].

Despite these significant improvements in access to SAC, it is unknown whether the public health sector is meeting the demand for safe abortion services. An important question that remains unanswered is how to motivate women still having abortions outside of facilities to access safe and legal care in health facilities. The 2014 abortion incidence and morbidity study indicated that a significant proportion of abortion takes place outside of facilities with mild to severe complications [9, 13]. This may indicate that many women are still not accessing safe abortions in facilities, likely due to barriers to access, limited knowledge of services and legality, and abortion-related stigma in the communities. With the revised technical and procedural guidelines, there are greater opportunities to get legal abortion care closer to women in communities in some of the far-reaching regions, through lower levels of facilities and health care workers including referrals from health extension workers (HEWs). For example, as one of the task shifting and task sharing area emphasized in the guideline, HEWs will support CAC service provision by assessing gestational age and providing referrals to health facilities. A pilot intervention in the Tigray region of Ethiopia in 2011 proved this approach to be a feasible and effective strategy [20]. Moreover, a recent study of the acceptability of the involvement of HEWs in medical abortion in two zones of the Oromia region showed that abortion clients and service providers accepted the involvement of HEWS in providing medical abortion, with HEWs expressing their readiness and confidence to provide this service [21]. A multi-country study of the accuracy of assessment of eligblity for early medical abortion by community health workers in Ethiopia, India and South Africa also showed the accuracy of the assessments by community health workers was over 90% in Ethiopia [22]. Thus, scaling up interventions that train HEWs and other lower-level health care workers to be part of CAC treatment and referrals could have a huge impact on increasing points of access to care.

There are several limitations to the SAC model for measuring progress in safe abortion access, utilization, and quality of care. The primary limitation is geographical. In larger regions with widely dispersed, hard to reach populations, it might be more useful to look at population density and distance to facilities as opposed to total regional population. Using population totals alone means that some regions require very few hospitals according to the SAC model, but they are very far away from a large proportion of the population. The findings concerning geographic distribution using the SAC model are therefore limited. A second limitation of the SAC model is its inability to measure many aspects of quality of care, such as the provision of medication for pain management. Using this model alone provides little information on women’s experiences and satisfaction with the services, which could be a facilitating factor or barrier to accessing care at facilities.

## Conclusion

This is one of very few studies to assess the impact of abortion policy reform and practice over time in a low-resource setting. Overall, the MoH’s strong commitment to the Scale-up of safe abortion services after legal reform has made significant progress toward making safe abortion care available for every woman in Ethiopia. The use of appropriate technology for conducting first and second trimester abortion and the provision of post abortion family planning has increased at the same time that abortion-related obstetric complications have decreased. Future SAC interventions should prioritize training providers to perform first and second trimester induced abortion, including the introduction of medical abortion and dilatation and evacuation (D & E) in hospitals to make second trimester abortion services available to women who need them. Increasing provision of comprehensive SAC services, particularly in large, remote regions is essential. Finally, there needs to be a renewed emphasis on community interventions that aim to decrease abortion stigma and increase residents’ knowledge of service availability in order to increase utilization of SAC services in facilities and thereby prevent unsafe abortion.

## Abbreviations

CAC: Comprehensive abortion care
CSA: Central Statistical Agency
D & E: Dilation and evacuation
EMOC: Emergency obstetric care
HEW: Health extension worker
HFS: Health Facility Survey
HPS: Health Professional Survey
MA: Medical abortion
MOH: Ministry of Health
MVA: Manual vacuum aspiration
NGO: Non-Governmental organizations
PAC: Post abortion care
PMS: Prospective Morbidity Survey
SAC: Safe abortion care
TOP: Termination of pregnancy
UE: Uterine evacuation
WHO: World Health Organization
